# High incidence and increasing prevalence of multiple sclerosis in British Columbia, Canada: findings from over two decades (1991–2010)

**DOI:** 10.1007/s00415-015-7842-0

**Published:** 2015-07-24

**Authors:** Elaine Kingwell, Feng Zhu, Ruth Ann Marrie, John D. Fisk, Christina Wolfson, Sharon Warren, Joanne Profetto-McGrath, Lawrence W. Svenson, Nathalie Jette, Virender Bhan, B. Nancy Yu, Lawrence Elliott, Helen Tremlett

**Affiliations:** Faculty of Medicine (Neurology), UBC Hospital, University of British Columbia, 2211 Wesbrook Mall, Vancouver, BC V6T 2B5 Canada; Departments of Internal Medicine & Community Health Sciences, University of Manitoba, Winnipeg, MB Canada; Departments of Psychiatry and Medicine, Dalhousie University, Halifax, NS Canada; Department of Medicine and of Epidemiology, Biostatistics and Occupational Health, McGill University, Montreal, QC Canada; Faculty of Rehabilitation Medicine, University of Alberta, Edmonton, AB Canada; Faculty of Nursing, University of Alberta, Edmonton, AB Canada; Surveillance and Assessment Branch, Alberta Health, Government of Alberta, Edmonton, AB Canada; Department of Clinical Neurosciences and Community Health Sciences, Hotchkiss Brain Institute and O’Brien Institute for Population Health, University of Calgary, Calgary, AB Canada; Department of Medicine (Neurology), Dalhousie University, Halifax, NS Canada; Department of Community Health Sciences, University of Manitoba, Winnipeg, MB Canada; Departments of Community Health Sciences and Medical Microbiology, University of Manitoba, Winnipeg, MB Canada

**Keywords:** Multiple sclerosis, Incidence, Prevalence, Epidemiology, Sex ratio, Administrative health data

## Abstract

Province-wide population-based administrative health data from British Columbia (BC), Canada (population: approximately 4.5 million) were used to estimate the incidence and prevalence of multiple sclerosis (MS) and examine potential trends over time. All BC residents meeting validated health administrative case definitions for MS were identified using hospital, physician, death, and health registration files. Estimates of annual prevalence (1991–2008), and incidence (1996–2008; allowing a 5-year disease-free run-in period) were age and sex standardized to the 2001 Canadian population. Changes over time in incidence, prevalence and sex ratios were examined using Poisson and log-binomial regression. The incidence rate was stable [average: 7.8/100,000 (95 % CI 7.6, 8.1)], while the female: male ratio decreased (*p* = 0.045) but remained at or above 2 for all years (average 2.8:1). From 1991–2008, MS prevalence increased by 4.7 % on average per year (*p* < 0.001) from 78.8/100,000 (95 % CI 75.7, 82.0) to 179.9/100,000 (95 % CI 176.0, 183.8), the sex prevalence ratio increased from 2.27 to 2.78 (*p* < 0.001) and the peak prevalence age range increased from 45–49 to 55–59 years. MS incidence and prevalence in BC are among the highest in the world. Neither the incidence nor the incidence sex ratio increased over time. However, the prevalence and prevalence sex ratio increased significantly during the 18-year period, which may be explained by the increased peak prevalence age of MS, longer survival with MS and the greater life expectancy of women compared to men.

## Background

Multiple sclerosis (MS), a chronic, debilitating disease of the central nervous system, is the leading cause of non-traumatic disability in young adults [1]. It is estimated that more than two million people live with this disease worldwide [1], although the incidence and prevalence vary geographically [2–4]. Furthermore, reports of recent increases in the incidence and prevalence, and in the ratio of women to men with MS, have been inconsistent across regions [5–7].

The need for current and reliable estimates of MS incidence and prevalence has been highlighted as a public health and research priority, essential to support the planning and prioritization of health care services and to reduce the overall burden of chronic disease [1, 8, 9].

Valid and reliable methods are required when estimating incidence and prevalence so that regional estimates can be compared. Validated case definitions that use population-based administrative data offer this opportunity where such data are available. Canada has a universal publicly funded health care system and, in its western-most province British Columbia (BC), health claims data for the entire population are captured. No estimates of the prevalence of MS in BC using population-based linked health administrative data have been reported, and the incidence of MS in BC has not been previously estimated by any method.

We aimed to estimate the incidence and prevalence of MS in BC, Canada using previously validated case definitions of MS [10, 11] based on health administrative data. Also, we described the demographics of the incident and prevalent cases and temporal changes in their characteristics including the sex ratio.

## Methods

British Columbia is situated on the west coast of Canada. Its population of 4.5 million people represents 13 % of the Canadian population. The publicly funded provincial health care programme is compulsory for residents; a lifelong unique personal health care number is assigned and is linked through provincial administrative databases to all hospital admissions, physician visits, prescription dispensations, births, deaths, and health care plan registration and cancellation dates.

Anonymized linked BC health administrative data files used in this study included the Hospital Admission and Discharge database (hospital admission dates and diagnosis codes) [12], and the Medical Services Plan Billing (physician visits and billing diagnosis codes) [13], These databases store data on physician billing or medical services claims (‘claims’) that have been submitted for payment, including the type of service provided, when and to whom the service was provided, and the diagnoses related to the physician visit or hospital admission (coded via International Classification of Disease (ICD-9 or ICD-10-CA)). PharmaNet (dispensed prescriptions coded by Health Canada’s Drug Identification Numbers) [14], and Vital Statistics (death dates) [15] were also accessed. Registration Premium and Billing files [16] provided demographic data: registration dates in the provincial health care plan confirmed residency in BC; socioeconomic status (SES) was expressed as quintiles of average neighbourhood income based on regional income levels (prepared by Statistics Canada using postal codes [17]).

We utilized a previously linked data platform which included all residents of BC with ≥3 MS-related claims. Linked data were available from 1986, apart from ICD codes from physician visits which were available starting in 1991. Prescription data were accessed for descriptive purposes only, and were available starting in 1996. Follow-up continued to the end of 2010 for the majority of those in the dataset, with the remainder followed to the end of 2008.

MS cases were identified using administrative case definitions, which have previously been validated in two Canadian provinces (Manitoba and Nova Scotia) [10, 11], and are based on hospital and physician-derived diagnostic codes. The primary case definition used was ≥7 hospital or physician claims specifically for MS for people who were resident in BC for >3 years following their first demyelinating disease (‘MS-related’) claim (i.e. a claim for MS, optic neuritis, acute transverse myelitis, acute disseminated encephalomyelitis, demyelinating disease of the CNS unspecified, other acute disseminated demyelination, or neuromyelitis optica), and ≥3 MS claims for those with ≤3 years of residency. When validated against the clinical MS definition, this algorithm was found to provide the best balance of sensitivity and specificity compared to a series of alternative administrative case definitions. For the validation population (Nova Scotia, Canada), all of whom had at least one claim for a demyelinating disease, this definition had a sensitivity of 88 % and specificity of 68 %; the specificity would, however, be substantially higher in the general population given that >99.9 % of individuals have no demyelinating claims [11, 18]. A second case definition was also used which required ≥3 MS claims irrespective of the cumulative residency in BC, for which previous validation has demonstrated greater sensitivity (95 %) but less specificity (48 %) among those with at least one demyelinating claim [11].

Point prevalence was estimated annually on July 1st, from 1991 to 2008, and incidence estimates were generated from 1996 to 2008, with inclusion of claims up to 2010. Both incidence and prevalence were calculated per 100,000 people using the BC mid-year population and were age and sex standardized to the 2001 Canadian population, for consistency to prior Canadian work [10, 11, 19]. The 95 % confidence intervals (CI) were calculated based on the Gamma distribution [20]. Incidence estimates began in 1996 because at least 5 years residency with no MS-related claim was required to meet the incident case definition. Once this definition was met, the date of the first MS-related claim was considered the incidence date of MS diagnosis. Individuals who immigrated to the province after the study start were followed from the date of their first registration in the universal BC health care plan; as with cases that were resident from study start, a 5-year residency with no MS-related claim was required to be counted as an incident case.

Description of the incident cases included sex, age, SES, time to meet the case definition, and dispensation of a MS disease-modifying drug (DMD) within 3 years of the incident claim. This time window was chosen because the diagnosis date falls within 3 years of the incident claim for approximately 75 % of MS cases [10]. Cases that were prevalent on July 1st 2008 were described by sex and age, SES and history of a DMD prescription (including beta interferon-1a, beta interferon-1b, glatiramer acetate and natalizumab). The distribution of cases across the SES quintiles was compared to the expected (even) distribution.

Changes in the incidence rate and prevalence over the observation period were investigated using Poisson (with the BC population included as an offset) and log-binomial regression, respectively. The models included an interaction term between year and sex. Potential differences in the distribution over socioeconomic quintiles were assessed using a Chi-Squared test of homogeneity.

Follow-up data for the years 2009 and 2010 were unavailable for some individuals who were alive and resident in BC at the end of 2008 but had not yet met the MS case definition. To assess the potential impact of this missing 2 years of follow-up data on the findings, the numbers of potentially missed incident and prevalent cases were calculated by assuming every individual with missing follow-up data would have met the case definition and the estimates and comparisons were repeated.

Statistical analyses were performed using R: A Language and Environment for Statistical Computing v.2.15 (R Foundation for Statistical Computing, Vienna, Austria; 2012).

This study was approved by the University of British Columbia’s Clinical Research Ethics Board (approval # H10-01361). BC Ministry of Health, BC Vital Statistics Agency and BC PharmaNet approved access to administrative health data.

## Results

Between 1996 and 2008, 4,222 BC residents met the incident case definition of ≥7 MS claims (or ≥3 MS claims for those with ≤3 years of residency in BC) and at least 5 years of residency before their first MS-related claim. The standardized annual incidence rate was 7.8 (95 % CI 7.6, 8.1) per 100,000 people; 11.5 (95 % CI 11.1, 11.9) for women and 4.1 (95 % CI 3.8, 4.3) for men. The more sensitive case definition of ≥3 MS claims identified 5876 incident cases for a standardized annual incidence estimate of 10.9 (95 % CI 10.6, 11.2) per 100,000.

Characteristics of the incident cases are summarized in Table 1; the sex, age and SES distributions were similar regardless of the case definition used. Women made up approximately three quarters of the incident cases. The overall mean age at the first MS-related claim was lower for women (44 years) than for men (46 years) (*p* < 0.001). The distribution of cases across the SES quintiles differed at the time of the incident claim, with more cases in the middle and higher SES quintiles, but the absolute differences were small. Among all incident cases (using the primary definition), 27 % filled a prescription for a DMD within 3 years of their incident claim; this proportion increased between 1996 and 2000 from 10 to 32 %; the proportion with prescriptions within 3 years then remained stable at 31–33 % from 2000 to 2007 (the last calendar year with 3 years of follow-up).

**Table 1:** Characteristics of the incident (1996–2008) and prevalent (2008) multiple sclerosis cases in British Columbia, Canada

Incident cases (1996–2008)	Primary definition *n* = 4222	3 Claims definition *n* = 5876
Sex, *n* (%)		
Women	3124 (74)	4315 (73)
Men	1098 (26)	1561 (27)
Age at incidence, years		
Mean (SD)	44.3 (13.2)	44.7 (13.4)
Median (1st quartile, 3rd quartile)	43.4 (35.2, 51.7)	43.8 (35.6, 52.4)
Time to meet case definition, years		
Mean (SD)	2.1 (2.2)	1.0 (1.7)
Median (1st quartile, 3rd quartile)	1.3 (0.5, 2.9)	0.4 (0.1, 1.1)
Prescription for a DMD, *n* (%)		
Ever	1411 (33)	1432 (24)
Within 3 years of incident claim	1143 (27)	1156 (20)
SES quintile at incident claim, *n* (%)^a^		
Lowest	759 (18)*	1050 (18)*
Second lowest	766 (18)	1091 (19)
Middle	902 (21)	1228 (21)
Second highest	857 (20)	1209 (21)
Highest	842 (20)	1163 (20)
Unknown	96 (2)	135 (2)

Prevalent cases (July 1st 2008)	Primary definition *n* = 8546	3 Claims definition *n* = 11,184
Sex, *n* (%)		
Women	6313 (74)	8206 (73)
Men	2233 (26)	2978 (27)
Age, years		
Mean (SD)	52.3 (12.6)	52.3 (12.9)
Median (1st quartile, 3rd quartile)	52.3 (43.8, 60.5)	52.1 (43.7, 60.5)
Prescription of a DMD, *n* (%)		
Ever	2485 (29)	2516 (22)
SES quintile, *n* (%)^a^		
Lowest	1541 (18)*	2028 (18)*
Second lowest	1612 (19)	2090 (19)
Middle	1745 (20)	2257 (20)
Second highest	1782 (21)	2352 (21)
Highest	1727 (20)	2272 (20)
Missing	139 (2)	185 (2)

*SD* standard deviation, *DMD* disease-modifying drug, *SES* socioeconomic status

* p ≤ 0.001

^a^Percentages may not sum to 100 due to rounding. Chi-squared test of homogeneity was used to compare SES quintiles to an expected equal distribution across the quintiles (cases with missing SES were excluded)

The median number of years between the first MS-related claim and fulfilling criterion for the primary case definition was 1.3 years overall; 1.2 years for women and 1.0 years for men. As the follow-up time decreased over the observation period, the median time to reach criterion naturally decreased; the longest was 2.1 years (interquartile range: 0.8, 5.3) in 1996 when up to 15 years of follow-up data were available. Using the more sensitive case definition, 75 % of cases reached criterion in 1.8 years (median 0.4 years) when up to 15 years of data were available.

**Fig. 1:**
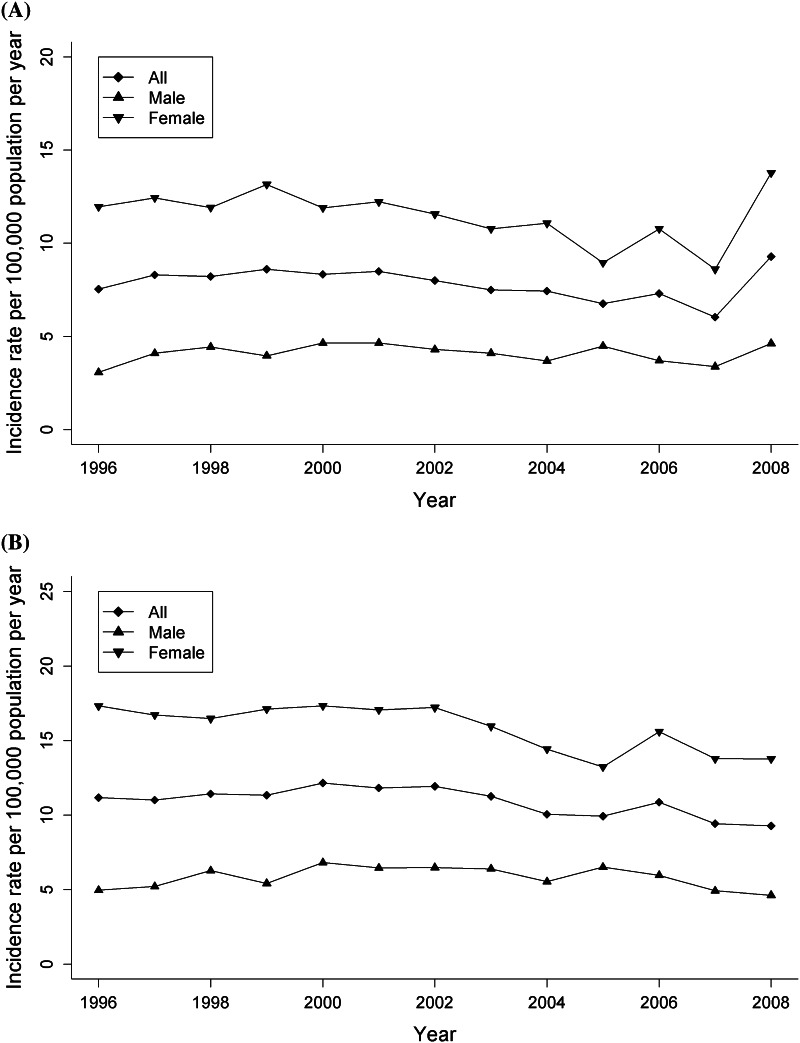
Age-standardized annual incidence rates (1996–2008) of multiple sclerosis cases identified by the primary case definition (**a**) and the more sensitive but less specific case definition (**b**) in British Columbia, Canada. Note: The apparent increased incidence in the final year observed in **a** is a result of the case criterion; all potential incident cases for that year had ≤3 years of follow-up available to study end and therefore required only 3 claims to meet case definition

Although there were small fluctuations in the annual incidence rate (Fig. 1a, b), there was no evidence of an increase in incidence between 1996 and 2008 regardless of the case definition used. The average female to male incidence ratio across all years was 2.8 (95 % CI 2.6, 3.0). This ratio varied by calendar year; the interaction between sex and year was statistically significant (*p* = 0.045) with a small decrease in the incidence rate in women, while the rate remained stable in men (Table 2).

**Table 2:** Number of incident cases and incidence rate of multiple sclerosis per 100,000 population per year (1996–2008) in British Columbia, Canada by sex and calendar year

Year	Women		Men		All		Incidence sex ratio (95 % CI)^a^
	Cases/popul.	Crude IR (95 % CI) *[standardized IR]*	Cases/popul.	Crude IR (95 % CI) *[standardized IR]*	Cases/popul.	Crude IR (95 % CI) *[standardized IR]*	
1996	231/1,944,984	11.9 (10.4, 13.5) *[12.0 (10.5, 13.6)]*	57/1,929,333	3.0 (2.2, 3.8) *[3.1 (2.3, 4.0)]*	288/3,874,317	7.4 (6.6, 8.3) *[7.5 (6.7, 8.5)]*	4.0 (3.0, 5.4)
1997	246/1,983,109	12.4 (10.9, 14.1) *[12.4 (10.9, 14.1)]*	79/1,965,474	4.0 (3.2, 5.0) *[4.1 (3.2, 5.1)]*	325/3,948,583	8.2 (7.4, 9.2) *[8.3 (7.4, 9.3)]*	3.1 (2.4, 4.0)
1998	238/2,002,595	11.9 (10.4, 13.5) *[11.9 (10.5, 13.5)]*	87/1,980,518	4.4 (3.5, 5.4) *[4.4 (3.6, 5.5)]*	325/3,983,113	8.2 (7.3, 9.1) *[8.2 (7.4, 9.2)]*	2.7 (2.1, 3.5)
1999	269/2,018,796	13.3 (11.8, 15.0) *[13.2 (11.6, 14.8)]*	79/1,992,579	4.0 (3.1, 4.9) *[4.0 (3.1, 4.9)]*	348/4,011,375	8.7 (7.8, 9.6) *[8.6 (7.7, 9.6)]*	3.4 (2.6, 4.3)
2000	246/2,033,853	12.1 (10.6, 13.7) *[11.9 (10.5, 13.5)]*	94/2,005,377	4.7 (3.8, 5.7) *[4.7 (3.8, 5.7)]*	340/4,039,230	8.4 (7.6, 9.4) *[8.3 (7.5, 9.3)]*	2.6 (2.0, 3.3)
2001	255/2,053,217	12.4 (10.9, 14.0) *[12.2 (10.8, 13.8)]*	95/2,023,047	4.7 (3.8, 5.7) *[4.7 (3.8, 5.7)]*	350/4,076,264	8.6 (7.7, 9.5) *[8.5 (7.6, 9.4)]*	2.6 (2.1, 3.4)
2002	243/2,066,320	11.8 (10.3, 13.3) *[11.6 (10.2, 13.1)]*	89/2,031,858	4.4 (3.5, 5.4) *[4.3 (3.5, 5.3)]*	332/4,098,178	8.1 (7.3, 9.0) *[8.0 (7.2, 8.9)]*	2.7 (2.1, 3.4)
2003	228/2,079,214	11.0 (9.6, 12.5) *[10.8 (9.4, 12.3)]*	85/2,043,182	4.2 (3.3, 5.1) *[4.1 (3.3, 5.1)]*	313/4,122,396	7.6 (6.8, 8.5) *[7.5 (6.7, 8.4)]*	2.6 (2.1, 3.4)
2004	235/2,096,756	11.2 (9.8, 12.7) *[11.1 (9.7, 12.6)]*	76/2,058,414	3.7 (2.9, 4.6) *[3.7 (2.9, 4.6)]*	311/4,155,170	7.5 (6.7, 8.4) *[7.4 (6.6, 8.3)]*	3.0 (2.3, 3.9)
2005	194/2,117,446	9.2 (7.9, 10.6) *[9.0 (7.7, 10.3)]*	96/2,079,342	4.6 (3.7, 5.6) *[4.5 (3.6, 5.5)]*	290/4,196,788	6.9 (6.1, 7.8) *[6.8 (6.0, 7.6)]*	2.0 (1.6, 2.5)
2006	235/2,141,450	11.0 (9.6, 12.5) *[10.8 (9.4, 12.3)]*	81/2,102,130	3.9 (3.1, 4.8) *[3.7 (2.9, 4.6)]*	316/4,243,580	7.5 (6.7, 8.3) *[7.3 (6.5, 8.2)]*	2.9 (2.2, 3.7)
2007	193/2,173,994	8.9 (7.7, 10.2) *[8.6 (7.4, 9.9)]*	73/2,135,530	3.4 (2.7, 4.3) *[3.4 (2.6, 4.3)]*	266/4,309,524	6.2 (5.5, 7.0) *[6.0 (5.3, 6.8)]*	2.6 (2.0, 3.4)
2008^b^	311/2,210,657	14.1 (12.6, 15.7) *[13.8 (12.3, 15.4)]*	107/2,173,653	4.9 (4.0, 6.0) *[4.6 (3.8, 5.6)]*	418/4,384,310	9.5 (8.6, 10.5) *[9.3 (8.4, 10.2)]*	2.9 (2.3, 3.6)
1996–2008	3124/26,922,391	11.6 (11.2, 12.0) *[11.5 (11.1, 11.9)]*	1098/26,520,437	4.1(3.9, 4.4) *[4.1 (3.8, 4.3)]*	4222/53,442,828	7.9 (7.7, 8.1) *[7.8 (7.6, 8.1)]*	2.8 (2.6, 3.0)

*IR* incidence rate, *Popul.* population (denominator), *CI* confidence interval

^a^Crude incidence sex ratio (incidence rate in women: incidence rate in men)

^b^The apparent increase in incidence in 2008 is due to the case criterion; as of 2008 all potential cases required only 3 claims, unlike in previous years, because ≤3 years of follow-up remained

On July 1st 2008, there were 8546 people with MS living in BC and the standardized prevalence per 100,000 people was 179.9 (95 % CI 176.0, 183.8). The prevalence estimates for each year by sex and the sex ratio for 1991–2008 are shown in Table 3. With the more sensitive case definition, the standardized prevalence on July 1st 2008 was 235.8 (95 % CI 231.4, 240.3), and an estimated 11,184 cases were living in BC on the point prevalence date.

**Table 3:** Number of prevalent cases and prevalence of multiple sclerosis per 100,000 population on July 1st (1991–2008) in British Columbia, Canada by sex and calendar year

Year	Women		Men		All		Prevalence sex ratio (95 % CI)^a^
	Cases/popul.	Crude PP (95 % CI) *[standardized PP]*	Cases/popul.	Crude PP (95 % CI) *[standardized PP]*	Cases/popul.	Crude PP (95 % CI) *[standardized PP]*	
1991	1731/1,692,156	102.3 (97.5, 107.2) *[108.8 (103.7, 114.2)]*	757/1,681,631	45.0 (41.9, 48.3) *[48.7 (45.3, 52.4)]*	2488/3,373,787	73.8 (70.9, 76.7) *[78.8 (75.7. 82.0)]*	2.3 (2.1, 2.5)
1992	2336/1,741,163	134.2 (128.8, 139.7) *[142.3 (136.5, 148.2)]*	964/1,727,639	55.8 (52.3, 59.4) *[59.8 (56.1, 63.7)]*	3300/3,468,802	95.1 (91.9, 98.4) *[101.1 (97.7, 104.7)]*	2.4 (2.2, 2.6)
1993	2701/1,790,843	150.8 (145.2, 156.6) *[158.7 (152.8, 164.9)]*	1092/1,776,929	64.5 (57.9, 65.2) *[65.3 (61.4, 69.3)]*	3793/3,567,772	106.3 (103.0, 109.8) *[112.1 (108.6, 115.8)]*	2.5 (2.3, 2.6)
1994	3011/1,843,834	163.3 (157.5, 169.2) *[170.3 (164.3, 176.6)]*	1195/1,832,241	65.2 (61.6, 69.0) *[68.8 (65.0, 72.9)]*	4206/3,676,075	114.4 (111.0, 117.9) *[119.8 (116.1, 123.5)]*	2.5 (2.3, 2.7)
1995	3288/1,893,063	173.7 (167.8, 179.7) *[179.8 (173.6, 186.1)]*	1286/1,884,327	68.3 (64.6, 72.1) *[71.4 (67.5, 75.4)]*	4574/3,777,390	121.1 (117.6, 124.7) *[125.7 (122.1, 129.4)]*	2.5 (2.4, 2.7)
1996	3535/1,944,984	181.8 (175.8, 187.8) *[187.1 (180.9, 193.4)]*	1359/1,929,333	70.4 (66.7, 74.3) *[73.2 (69.3, 77.2)]*	4894/3,874,317	126.3 (122.8, 129.9) *[130.4 (126.8, 134.1)]*	2.6 (2.4, 2.8)
1997	3841/1,983,109	193.7 (187.6, 199.9) *[197.8 (191.6, 204.2)]*	1432/1,965,474	72.9 (69.1, 76.7) *[74.9 (71.1, 78.9)]*	5273/3,948,583	133.5 (130.0, 137.2) *[136.6 (133.0, 140.4)]*	2.7 (2.5, 2.8)
1998	4048/2,002,595	202.1 (196.0, 208.5) *[204.5 (198.2, 210.9)]*	1514/1,980,518	76.4 (72.6, 80.4) *[77.5 (73.6, 81.5)]*	5562/3,983,113	139.6 (136.0, 143.4) *[141.3 (137.6, 145.1)]*	2.6 (2.5, 2.8)
1999	4325/2,018,796	214.2 (207.9, 220.7) *[214.4 (208.0, 220.9)]*	1596/1,992,579	80.1 (76.2, 84.1) *[80.1 (76.2, 84.1)]*	5921/4,011,375	147.6 (143.9, 151.4) *[147.7 (144.0, 151.5)]*	2.7 (2.5, 2.8)
2000	4563/2,033,853	224.4 (217.9, 231.00) *[222.3 (215.9, 228.9)]*	1672/2,005,377	83.4 (79.4, 87.5) *[82.3 (78.4, 86.4)]*	6235/4,039,230	154.4 (150.6, 158.2) *[152.9 (149.1, 156.7)]*	2.7 (2.5, 2.9)
2001	4806/2,053,217	234.1 (227.5, 240.8) *[230.0 (223.5, 236.6)]*	1761/2,023,047	87.1 (83.0, 91.2) *[85.1 (81.2, 89.2)]*	6567/4,076,264	161.1 (157.2, 165.0) *[158.2 (154.4, 162.0)]*	2.7 (2.6, 2.8)
2002	5086/2,066,320	246.1 (239.4, 253.0) *[239.6 (233.1, 246.3)]*	1846/2,031,858	90.9 (86.8, 95.1) *[87.8 (83.8, 91.9)]*	6932/4,098,178	169.2 (165.2, 173.2) *[164.4 (160.6, 168.3)]*	2.7 (2.6, 2.9)
2003	5285/2,079,214	254.2 (247.4, 261.1) *[244.9 (238.4, 251.6)]*	1925/2,043,182	94.2 (90.1, 98.5) *[90.1 (86.1, 94.2)]*	7210/4,122,396	174.9 (170.9, 179.0) *[168.3 (164.4, 172.3)]*	2.7 (2.6, 2.8)
2004	5490/2,096,756	261.8 (255.0, 268.8) *[249.8 (243.2, 256.5)]*	1962/2,058,414	95.3 (91.2, 99.6) *[90.2 (86.2, 94.3)]*	7452/4,155,170	179.3 (175.3, 183.5) *[170.9 (167.0, 174.8)]*	2.8 (2.6, 2.9)
2005	5705/2,117,446	269.4 (262.5, 276.5) *[254.3 (247.7, 261.0)]*	2044/2,079,342	98.3 (94.1, 102.7) *[92.0 (88.1, 96.1)]*	7749/4,196,788	184.6 (180.6, 188.8) *[174.1 (170.2, 178.0)]*	2.7 (2.6, 2.9)
2006	5891/2,141,450	275.1 (268.1, 282.2) *[257.5 (250.9, 264.2)]*	2097/2,102,130	99.8 (95.5, 104.1) *[92.5 (88.6, 96.6)]*	7988/4,243,580	188.2 (184.1, 192.4) *[176.0 (172.1, 179.9)]*	2.8 (2.6, 2.9)
2007	6073/2,173,994	279.4 (272.4, 286.5) *[259.9 (253.3, 266.6)]*	2143/2,135,530	100.4 (96.2, 104.7) *[92.3 (88.3, 96.3)]*	8216/4,309,524	190.7 (186.6, 194.8) *[177.0 (173.2, 180.9)]*	2.8 (2.7, 2.9)
2008	6313/2,210,657	285.6 (278.6, 292.7) *[264.0 (257.5, 270.7)]*	2233/2,173,653	102.7 (98.5, 107.1) *[93.9 (90.0, 97.9)]*	8546/4,384,310	194.9 (190.8, 199.1) *[179.9 (176.0, 183.8)]*	2.8 (2.7, 2.9)

*PP* point prevalence, *Popul.* population (denominator), *CI* confidence interval

^a^Crude prevalence sex ratio (prevalence proportion in women: prevalence proportion in men)

The average age of the 8546 prevalent cases was 52 years and 74 % were women. As observed for the incident cases, a comparison across the SES groups revealed an uneven distribution of prevalent MS cases with more prevalent MS cases in the higher quintiles of SES than in the lower quintiles, but small absolute differences. Sex, age and SES distributions were similar for the prevalent cases identified by the alternative administrative case definitions (Table 1). At least 29 % of the prevalent cases had received a prescription for a DMD at some point during their follow-up (or 22 % of cases identified by the more sensitive definition).

**Fig. 2:**
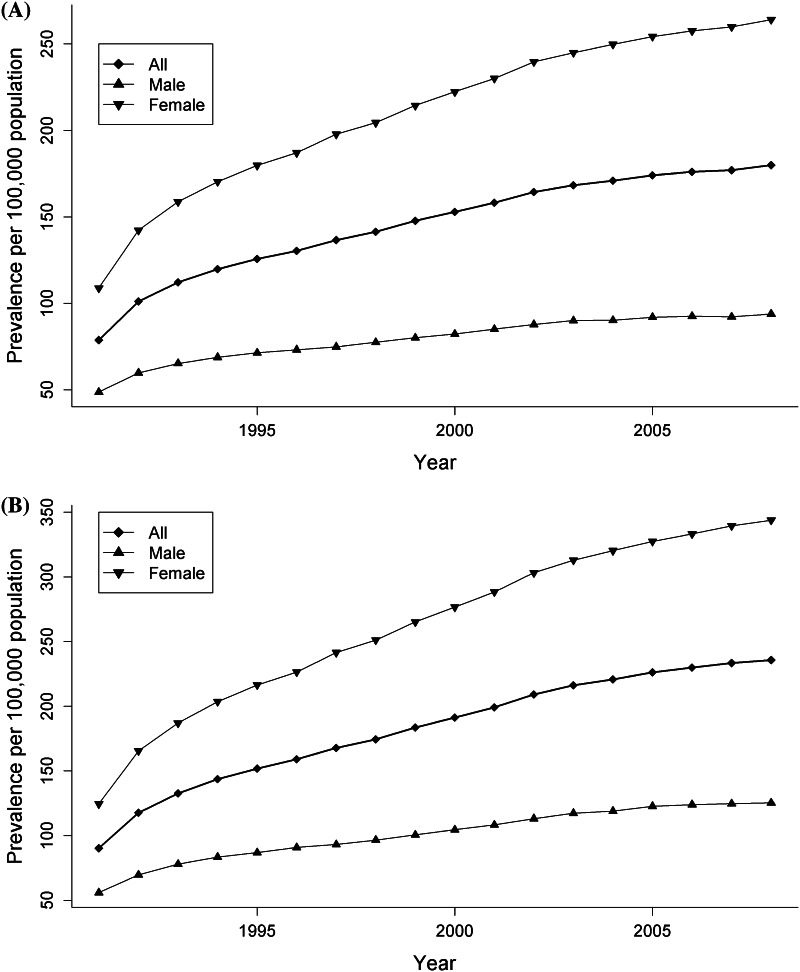
Age-standardized prevalence (1991–2008) of multiple sclerosis cases identified by the primary case definition (**a**) and the more sensitive but less specific case definition (**b**) in British Columbia, Canada

**Fig. 3:**
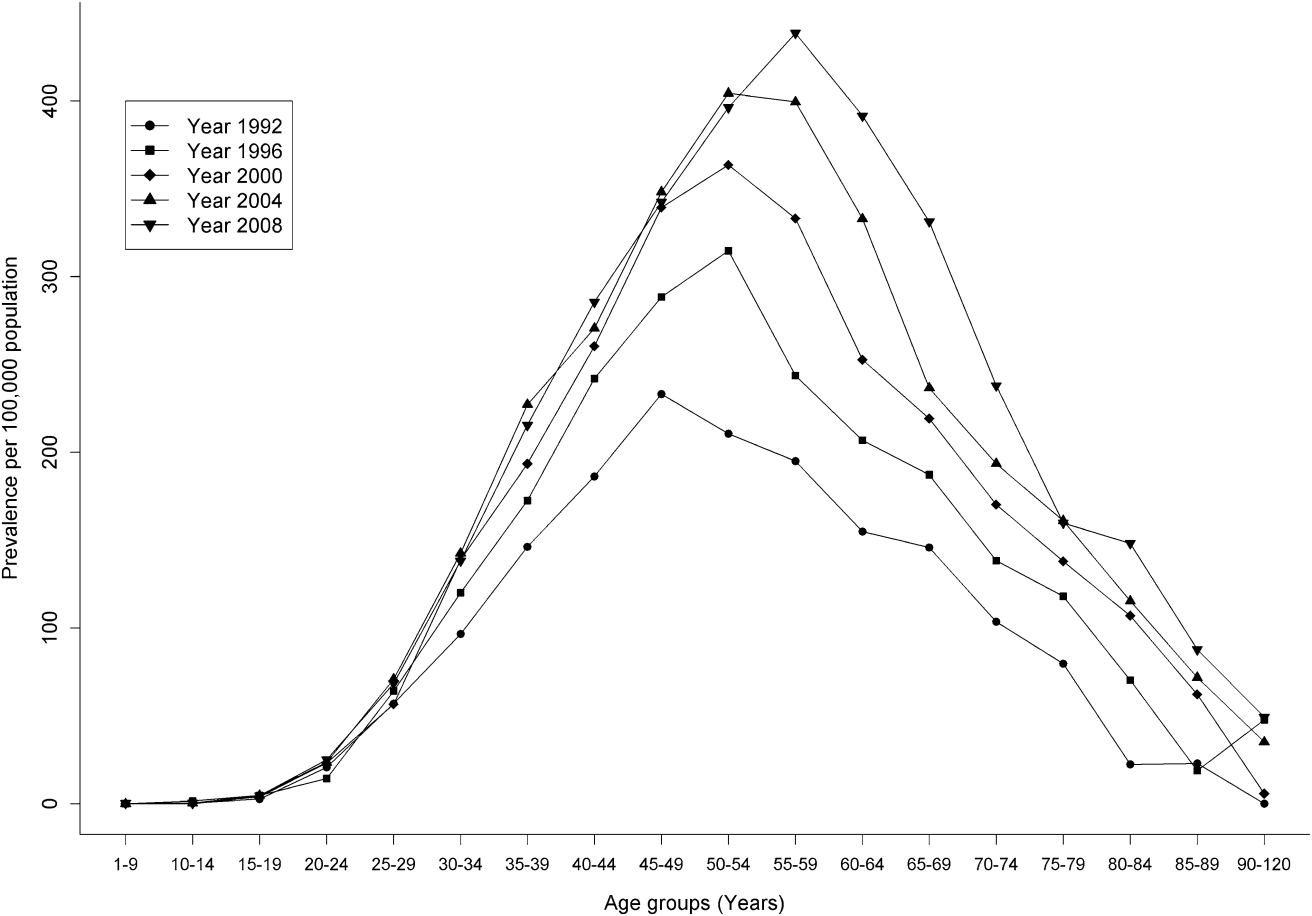
Age-specific prevalence of multiple sclerosis identified by the primary case definition per 100,000 population by select years (1992, 1996, 2000, 2004 and 2008) in British Columbia, Canada

The prevalence of MS increased over the 18-year observation period by an average of 4.7 % per year (*p* < 0.001), and the sex prevalence ratio increased from 2.27 in 1991 to 2.78 in 2008 (*p* < 0.001) (Fig. 2a, b). Overall, the peak age of prevalent MS cases increased over time from 45–49 years in the early 1990s to 55–59 years in 2008 (Fig. 3).

Among those with missing follow-up information for 2009–2010, there were 254 individuals with ≥1 demyelinating claim by the end of 2008 that had not yet met the primary case definition and 74 who had not yet met the more sensitive definition. These extra cases could potentially have increased the MS prevalence estimate for 2008, had complete follow-up data to 2010 been available. The maximum potential impact is an underestimate of the 2008 prevalence by up to 5.3/100,000 (or 1.6/100,000 using the ≥3 claim definition). The average annual 1996–2008 incidence may have been underestimated by up to 0.2 cases per 100,000, while the estimated incidence using the alternative case definition would not have been affected. When all of these potential cases were assumed to have met definition and included in the estimates and comparisons, all findings related to changes over time and characteristics of the incident and prevalent cases remained the same.

## Discussion

The prevalence of MS in BC, Canada has risen steadily and substantially, from 78.8/100,000 in 1991 to 179.9/100,000 in 2008. The incidence rate in BC remained stable over the study period, averaging 7.8 per 100,000 new cases of MS per year between 1996 and 2008, which is high relative to other parts of the world [1–4]. The prevalence sex ratio increased over time; however, the incidence sex ratio, which averaged 2.8:1, did not increase.

Methodological differences make direct comparisons with earlier studies of MS prevalence in BC difficult. Other than a study based on self-reported MS [21], the last province-wide estimate of MS prevalence, which used clinically confirmed definitions, was 93.3/100,000 in 1982 [22]. Our estimates for 1991 (78.8/100,000) and 1992 (101.1/100,000) are compatible with this estimate from 10 years earlier. There are no previous estimates of MS incidence in BC with which to compare our observations. However, using the same validated administrative MS case definitions age standardized to the same population, a similar annual incidence rate was recently found in central Canada [10] (11.4/100,000 in Manitoba using the more sensitive case definition). While a somewhat higher annual incidence was found in eastern Canada [11] (9.8/100,000 in Nova Scotia using our primary case definition), the 95 % confidence intervals overlapped with those in our study. BC, Manitoba and Nova Scotia are home to approximately 19 % of the Canadian population; extrapolating the combined estimate from these provinces (weighted by their relative population) to Canada, which had a population of 35.5 million in 2014, would mean that approximately 3000 new MS cases are diagnosed each year, or 8 new cases per day. Furthermore, extrapolating the combined prevalence estimate (200/100,000) from the three provinces would mean that approximately 71,000 people are living with MS in Canada.

Our observations that the prevalence of MS has been increasing by approximately 4.7 % per year in BC, and that the age of the prevalent population has risen have important implications for broader society, including governments and health care planners. We also found a gradual increase in the proportion of women to men living with MS; this is compatible with recent observations from elsewhere in Canada [10, 11] and the UK [23], and is likely due to the changing demographics (older mean age) of the general population and the greater life expectancy of women compared to men.

The rising prevalence in BC cannot be explained by increasing numbers of new MS cases; our incidence rates remained relatively stable over the 13-year period despite changes in MS diagnostic criteria [24] and increasing availability of disease-modifying drugs. While this seems in contrast to some other regions of the world where recent increases in incidence rates have been reported [5], a stable incidence rate has also been found over a similar time period in other Canadian provinces [10, 11, 19, 25, 26] and the UK [23]. Taken together with our findings, this suggests that the incidence of MS has stabilised in some areas over recent years. In the absence of increasing incidence, the rising prevalence may reflect longer disease duration due to earlier diagnosis, improved survival with MS or both. Survival has improved for both the BC general population and for people with MS in BC over the past 30 years [27]. Similarly, improved survival has been found in other MS populations, including those from Denmark [28] and Norway [29]. Immigration of prevalent cases can also influence prevalence trends and the population of British Columbia increased by nearly 30 % between 1991 and 2008, mostly due to immigration from other Canadian provinces and other countries. The prevalence estimates include MS cases that were resident throughout, as well as those that immigrated to BC, during the observation period. Newly immigrant prevalent cases would have contributed to the increasing prevalence over time if there was a greater proportion of MS cases among those immigrating to BC.

The average age at the time of the incident MS-related claim was 44 years. This age is comparable to that recorded in other Canadian provinces [10, 11] and was found to be within 3 years of the MS diagnosis date from medical charts or by personal report for 74 and 76 % of cases, respectively [10]. It is also equivalent to that identified as the first date of diagnosis in the General Practice Research Database for cases of MS in the UK [23]. While the date of diagnosis, or of first medical recognition, is frequently used to measure MS incidence [2–4], symptom onset can often occur several years before the disease is first recognized or diagnosed.

Notably, while others have reported increases in the incidence ratio of women to men with MS [6], we found no evidence of such a trend in BC. Rather, we observed a decrease in this ratio over time, although the absolute differences were small. Nonetheless, the more consistently observed sex differences for MS were evident; nearly three quarters of incident cases in BC were women and men were approximately 3 years older than women at the time of the first MS-related claim reflecting typical differences in onset age between sexes.

We observed a small difference in the distribution of cases across the socioeconomic quintiles, with a greater proportion of both incident and prevalent cases in the upper quintiles and fewer in the lower. Similar observations have been made in the past [30–34], although others have reported either no relationship or a negative association with SES [35].

Approximately, one-third of incident or prevalent MS cases filled a prescription for a DMD during the study period, stabilizing from the year 2000 onwards. This proportion may seem low, particularly when compared to a previous estimate (73–85 %) derived from a volunteer sample of patients recruited from Canadian MS treatment centres [36]. However, our proportion was derived from population-based rather than clinic-based data, the first DMD (interferon beta-1b) was only approved for use in Canada in 1995, and not all individuals with MS would have been eligible for treatment (including those unable to walk, those without relapses and those with a primary progressive disease course). Thus, it is likely that our data provide a realistic representation of DMD use in the British Columbian MS population over the study period.

The strengths of our study include the use of administrative health data, which represents the entire BC population, and spans nearly two decades allowing us to assess temporal trends. Furthermore, we used two previously validated MS case definitions to generate these estimates [10, 11]. The primary definition was identified as the best in terms of balance between specificity and sensitivity among candidate validated MS case definitions [11]. The secondary definition generated higher estimates due to its greater sensitivity, but may have included a greater proportion of false positives. Estimates generated by the primary definition could be more useful when it is important to minimize the risk of including people without MS, whereas the estimates from the more sensitive definition are likely more useful for estimating burden of disease and for health care planning. Although these MS case definitions were not validated specifically using the BC administrative data, the algorithms have been validated in Nova Scotia and Manitoba [10, 11]. Furthermore, similar 7-claim administrative case definitions of MS derived from administrative data in Ontario, Canada were validated in a primary care dataset in that province and found to have excellent performance [18]. The structure of the Canadian public health care system and the methods for coding physician and hospital visits in administrative health data are consistent between these three provinces and BC, which suggests that the case definitions would perform well and can be reliably applied in BC. The BC administrative health databases have been used, both independently and combined with equivalent data from other Canadian provinces, to identify and study other chronic diseases such as diabetes and hypertension [37–39].

Health administrative data have limitations. Although we allowed a five-year claim-free run-in period to capture incident cases, it remains possible that prevalent benign MS cases that rarely interacted with the medical system were misclassified as incident. Ascertainment is a common problem with MS incidence studies due to the inevitable time-lag between symptom onset and recognition of the disease; the estimated incidence may be affected by incomplete ascertainment towards the end of follow-up. Similarly, the prevalence estimates for the earliest years (1991 and 1992) should be treated cautiously; atypical prevalent cases with infrequent contact with the medical system could have been missed in these years. On the other hand, although we had missing follow-up data for up to 254 potential MS cases and could not confirm that they met criteria with follow-up to 2010, the estimates were not notably impacted.

We were unable to consider ethnicity, or country of origin. Although most BC residents are of European ancestry, BC has a higher proportion of residents of non-European origin than other Canadian provinces. The proportion with European ancestry has declined over time, from 82 % of the BC population in 1996 [40] to 75 % in 2006 [41]; people of Asian ancestry represent the largest minority group. The somewhat lower MS incidence rate in BC compared to that in Eastern Canada [11] might be explained by differences in the ethnic composition of the source populations [41].

In summary, BC has a high incidence of MS which has remained stable for more than a decade. However, as elsewhere in Canada, the prevalence and the peak age of the MS population have increased significantly. Population-based administrative health databases and validated case definitions of MS using health claims data provide a reliable, accessible and cost effective means of monitoring the incidence and prevalence of MS.
